# On the Action of Opium on the Genito-Urinary Organs

**Published:** 1863-05

**Authors:** 


### ON THE ACTION OF OPIUM ON THE GENITO-URINARY ORGANS.

The *Medical Times and Gazette*, of the 30th November, gives the following extract from Dr. Woodward's paper:—The author states that an accident led him to notice in his own person the incorrectness of the assertion so generally made, that opium arrests the urinary secretion. On the contrary, he found, by repeated experiments, that his urine, while taking small doses of sulphate of morphia, doubled in quantity and decreased in specific gravity from 1014 to 1003. Five young men were also experimented upon, and in four a large increase in quantity and a lower specific gravity were observed. In the other the quantity remained the same, but the specific gravity was markedly lessened. He did not obtain the same results when he used opium itself, but they followed whether he employed the muriate or sulphate of morphia. Acting upon this hint, he has several times used morphia in irritable conditions of the nervous system, where a diuretic was required, and with good effect. "I think it will be found, says he, in many cases of disease, the urinary secretion is arrested by the state of nervous tension which has been superinduced, and that, instead of a resort to stimulant diuretics, sedatives will relax the tension and allow the secretion to be restored." Another marked action of opium, Dr. Woodward observes, is as an anaphrodisiac. He has found that among opium eaters, both male and female, the sexual desire becomes almost extinct; while he has prescribed it with good effect in persons suffering from lustful propensities.

